# Functional Nervous Disease and the Courts of Law

**Published:** 1907-02-23

**Authors:** 


### The Hospital

A Journal of

The Medical Sciences and Hospital Administration.

Vol. XLI.—No. 1067. SATURDAY, FEBRUARY 23, 1907.

### Functional Nervous Disease and the Courts of Law.

Some of the possible ill-effects produced on the central nervous system by mechanical violence are almost as readily appreciated by the lay mind as by those who have enjoyed the advantages of a medical education. In an age of vigorous activities and multiple new applications of physical force, there are, unfortunately only too often, many opportunities for observing the disabling and destructive consequences which follow structural damage to the human body, and such a result as paralysis, for example, is widely recognised as a not unnatural issue from, say, a tramcar collision or a railway accident. Most members of the general public are roughly familiar with such an event, and recognise that injuries of this order afford an effective basis in a claim for compensation. As opposed to this, is the position in which injuries are simulated for the purpose of fraudulently establishing such a claim, and when the controversy in an individual case comes into the courts of law, the acute question usually is whether, as a matter of fact, the injuries are actual or pretended. Are the symptoms of which the plaintiff complains real, or are they manufactured for a purpose? If real, do they or do they not mean organic damage to the nervous system? These are the issues presented to the court and jury, and medical evidence is expected to supply simple, confident, and direct answers to the questions in dispute. Now, while there are cases in which special complications and complexities exist, it is not as a rule very difficult for a competent medical practitioner to decide whether there is or is not evidence of organic disease. There are certain definite signs of structural change the existence of which makes possible only a single interpretation, and the absence of these affords an extremely strong presumption in the opposite direction. Thus, to many, it appears as if the whole question of reasonable compensation for injury were contained in the narrowest possible compass.

A very different position, however, comes into view when it is recognised that severe and protracted interference with the functions of motion and sensation may follow the application of violence, even though no appreciable structural damage be inflicted on the nervous system. Whether the harm is produced through something in the nature of concussion, or as a consequence of mental disturbance, matters not. It is beyond dispute that traumatic neurasthenia exists and that its evidences may include events so eminently suggestive of structural damage to the nerve centres as loss of sensation and of the power of voluntary movement. Here, then, arises the difficulty. Actual damage is comprehensible; malingering is comprehensible; but who shall picture for a jury of laymen a state of matters in which, though extreme symptoms exist, there is neither organic disease on the one hand nor fraud on the other? Such a condition in its essential nature is not readily grasped even by the medical practitioner, and to the lay mind it is apt to appear frankly absurd. It is to be anticipated that, in most instances at all events, the lawyers and laymen who endeavour to dispense justice through our courts of law would make short work of such a doctrine. Yet the doctrine is undoubtedly true, and sooner or later it will have to receive legal countenance and recognition. A patient with, for example, functional hemiplegia as a sequel to, say, a railway accident, is as much the victim of a true paralysis as is a person who suffers from the effects of mechanical pressure on his motor pathway. The prospects of the two conditions may, perhaps, differ widely, and may need to be adjusted by individual balance and argument. But in each there is a real and true paralysis. Given that the two, more or less promptly, follow the application of violence, each may reasonably be described as the result of that violence.

From this it follows, that if such violence has been applied illegally, the person applying it may be held responsible for the paralysis, and this, equally, whether the paralysis be of organic or functional quality. It is not for us to decide what view the lawyers will take of this position; but without question the just definition is that here presented. And what we wish to urge is that medical practitioners shall be prepared to support it in open court. Terms like "hysterical," "malingering," and "functional" are, as the medical profession well knows, not synonymous. And in spite of the risk of misrepresentation, the profession must try to make the courts of law appreciate the difference. The task will not be an easy one, but justice demands that the attempt be made. In the United States a case has been successfully conducted in which the legal representatives of the plaintiff frankly admitted that the paralysis from which she suffered, as a sequel to a railway accident, was not organic but functional; and the court awarded heavy damages against the defendant company. Whether a British court will be equally ready to listen to the doctrine of functional disease has yet to be tested. But at the proper time we trust medical witnesses here, as on the other side of the Atlantic, will do their best to secure justice in this matter.

